# Impact of Sacubitril/Valsartan on Clinical and Echocardiographic Parameters in Heart Failure Patients With Reduced Ejection Fraction: Data From a Real Life 2-year Follow-Up Study

**DOI:** 10.3389/fphar.2021.733475

**Published:** 2021-08-16

**Authors:** Giuseppe Armentaro, Graziella D’Arrigo, Marcello Magurno, Alfredo F. Toscani, Valentino Condoleo, Sofia Miceli, Velia Cassano, Raffaele Maio, Franco Arturi, Giovanni Tripepi, Giorgio Sesti, Angela Sciacqua

**Affiliations:** ^1^ Department of Medical and Surgical Sciences, University Magna Græcia of Catanzaro, Catanzaro, Italy; ^2^ CNR-IFC, Istituto di Fisiologia Clinica, Clinical Epidemiology and Physiopathology of Renal Diseases and Hypertension, Reggio Calabria, Italy; ^3^ Department of Clinical and Molecular Medicine, University Rome-Sapienza, Rome, Italy

**Keywords:** heart failure, sacubitril/valsartan, cardiac index, left ventricular ejection fraction, NT-proBNP

## Abstract

Heart failure (HF) represents a widespread health problem characterized by high morbidity and mortality. Sacubitril/Valsartan (sac/val) has improved clinical prognosis in patients affected by HF with reduced ejection fraction (HFrEF). The aim of this study was to evaluate the effectiveness and durability of sac/val treatment on the clinical, hemodynamic and echocardiographic parameters, in real-life consecutive HFrEF outpatients, evaluated up to 2-years of follow-up. We collected 300 repeated observations over time in 60 patients suffering of HFrEF and symptomatic despite optimal drug therapy. Patients with left ventricular ejection fraction (LVEF) <35 and II-III NYHA functional class were considered. All patients underwent to clinical-instrumental and laboratory determinations and Minnesota Living with HF Questionnaire (MLHFQ) every 6 months until 24 months to evaluate possible clinical benefits and adverse events. During a 2-year follow-up period and through a 6-monthly control of the study variables both clinical, hemodynamic, biochemical and echocardiographic parameters significantly improved, in particular cardiac index (CI), both atrial and ventricular volumes and global longitudinal strain (GLS). Furthermore, there was a reduction of NT-proBNP levels and betterment of renal function and NYHA functional class, demonstrating the efficacy and durability of sac/val treatment. In a multiple linear mixed model the longitudinal evolutions of CI were associated to concomitant changes of GLS and E/e’ ratio. Our study, contemplating the collection of 300 repeated observations in 60 patients, provides a complete and detailed demonstration of sac/val effects, showing effectiveness, safety and effect durability of the treatment every 6 months up to 2-years of follow-up with significant improvement of several clinical, hemodynamic and echocardiographic parameters in HFrEF outpatients.

## Introduction

Heart failure (HF) represents a widespread health problem with an estimated prevalence in European countries of 1–2% in the adult population and over 10% in elderly (Ponikowski, et al., 2016; Mosterd A, Hoes AW, 2007). Despite important achievements in pharmacological treatment, HF is characterized by high morbidity and mortality. The Italian Network Heart Failure (IN-HF) registry showed that 1-year mortality rate was higher than 27% in patients who experienced worse clinical conditions and needed hospital admission (Tavazzi L, et al., 2013). In this context, HF with reduced ejection fraction (HFrEF) represents about a 50% of the whole affected population, and this is a fluctuating clinical condition characterized by phases of apparent clinical stability and clinical worsening which frequently require hospitalization (Iorio A, et al., 2019; Dharmarajan K, et al., 2013). Epidemiology data based on hospital admission showed that HF is the second cause of hospitalization for patients > 65 years, and despite optimal treatment, the patients’ prognosis after discharge remains poor. According with this, about 25% of hospitalized patients are readmitted within 30 days and a 50% of patients has a readmission in the next 6 months (Tavazzi L, et al., 2013; Dharmarajan K, et al., 2013). In addition, a clinically significant disease progression is observed in about 40% of patients, with a higher mortality in the advanced New York Heart Association (NYHA) class (Tavazzi L, et al., 2013; Iorio A, et al., 2019). All these observations imply a substantial increase in major costs for the National Healthcare System thus making HF an important health-care resource burden (Liao L, et al., 2008; Stewart S, et al., 2002). Even if renin-angiotensin-aldosterone system (RAAS) inhibitors and beta-blockers (BBs) represent the corner stone of pharmacological therapy for HFrEF, new therapeutic targets have been identified to improve clinical outcome. According with this, sacubitril/valsartan (sac/val) is an angiotensin receptor-neprilysin inhibitor (ARNI) approved in Italy since March 2017 that combines the reduced degradation of natriuretic peptides with selective AT1-receptor blockade favouring positive effects on cardiovascular (CV) system (Vardeny O, et al., 2014). The Prospective Comparison of ARNI with an ACE-Inhibitor to Determine Impact on Global Mortality and Morbidity in Heart Failure (PARADIGM-HF) trial demonstrated that sac/val was able to reduce the composite endpoint of CV death or first hospitalization for HF by 20% and the relative risk of all-cause mortality by 16% in comparison with enalapril, during a 27 months median of follow-up, in HRrEF outpatients. Moreover, the beneficial effect of sac/val was interestingly evident on the reduction of 30-days readmission and quality of life (McMurray JJ, et al., 2014; Vardeny O, et al., 2019) and the magnitude of the effect was similar both in patients without prior HF hospitalization and in more recently hospitalized patients (Solomon SD, et al., 2016). In addition, sac/val is more effective than enalapril also among patients hospitalized for acute decompensated heart failure (ADHF) in reducing both N-terminal pro-brain natriuretic peptide (NT pro-BNP) and, in an exploratory analysis, also the composite endpoint of rehospitalization and CV death (Velazquez EJ, et al., 2019; Morrow DA, et al., 2019).

However, real life setting may be largely different from clinical trials, and because sac/val is being increasingly prescribed, real world data analyses are needed. Emerging real world evidence show that sac/val treatment is associated with amelioration in functional class and exercise tolerance (Beltrán P, et al., 2018), improvement in cardiac remodelling and markers of cardiac volume and renal function (Almufleh A, et al., 2017; Januzzi JL, et al., 2019; Spannella F, et al., 2019) with reduction in hospital readmissions (Moliner-Abós C, et al., 2019). In real life setting the use of sac/val is also associated with high persistence and compliance (Wachter R, et al., 2019), however all these observations mostly refer to short terms treatment periods, and longer observations are needed. This is a crucial issue, because when a neurohormonal system is chronically blocked it is possible that other collateral systems may be activated thus impairing the efficacy of the pharmacological block (Singh JSS, et al., 2017). The aim of the present study was to evaluate, in a large series of 300 repeated observations in 60 patients, the effectiveness and durability of sac/val treatment on the clinical, hemodynamic and echocardiographic parameters, in real-life consecutive HFrEF outpatients, evaluated up to 2-years of follow-up.

## Materials and Methods

### Study Population

We performed a longitudinal, observational, one-center study, analyzing all clinical, laboratory and echocardiographic parameters of 60 consecutive HFrEF Caucasian outpatients totalizing 300 repeated observations over time. Patients were referred to the Geriatrics Department at the University Hospital of Catanzaro, and all underwent to sac/val treatment according to the International Guidelines recommendations (Ponikowski, et al., 2016). Thus, only adult subjects (age > 18 years) with FE ≤ 35%, in NYHA II or III class and with stable doses of angiotensin-converting enzyme inhibitor (ACE-I) or angiotensin receptor blocker (ARB) for at least 4 weeks but still symptomatic were considered for the analysis. The study population consisted in 52 men and eight females with an average age of 67 ± 11 years, followed between February 2017 and March 2020. No patient had clinical history of severe renal disease [estimated-glomerular filtration rate (e-GFR) <30 ml/min/1.73 m^2^] or hepatic impairment (Child-Pugh Class C), angioedema, side effects to ACE-I or ARB. None of patient was pregnant or breastfeeding, none of them had potassium levels >5.4 mmol/L or systolic blood pressure (SBP) < 100 mmHg. Patients referred for resynchronization therapy within 12 months before the enrolment and during the study, were excluded from analysis. All patients underwent an accurate medical history and a complete physical examination with the determination of the main anthropometric [weight, height, and body mass index (BMI)] and hemodynamic parameters. Moreover, NYHA functional class, quality of life (QoL) by the Minnesota Living with Heart Failure Questionnaire (MLHFQ), a 12-lead electrocardiogram (ECG) using a Philips PageWriter T10 electrocardiograph and a complete echocardiogram were also assessed. Ethics Committee approved the protocol (code protocol number 2012.63) and informed consent was obtained from all participants. All investigations were carried out to accordance with the principles of the Helsinki Declaration. BP measurements were made at the supine patient’s non-dominant arm after 5 minutes of rest. The values of the SBP and diastolic BP (DBP) were recorded, respectively, in the first (phase I) and last (phase V) tone of Korotkoff. Baseline BP values represent the average of three measurements obtained at 3 minute intervals.

Patients eligible for sac/val, in addition to their previous therapy, after suspension of ACE-I (at least 36 h before) or ARB, received initial dosage of 24/26 mg or 49/51 mg bid according to clinical parameters; the dosage was increased every 2–4 weeks up to the maximum tolerated dose. Clinical evaluation, laboratory tests, ECG and echocardiograms were evaluated at baseline [time 0 (T0)] and every 6 months up to 2 years (T6, T12, T18, T24) to estimate the possible benefits and the occurrence of any adverse events. Obviously, in addition to sac/val, all the other CV drug classes were also considered and their changes during the follow-up were analyzed.

### Echocardiographic Measurements

Echocardiographic examinations were performed with a monoplane ultrasound probe 2.5 MHz of Vivid E-95 (GE Medical Systems, Milwaukee, United States of America) by a single trained operator, who was blinded to treatment protocol. All patients were examined at rest and in the left lateral decubitus position, at the end of a normal breath, minimising the depth in order to optimise the frame rate (40–80 fps). The measurements were obtained according to the international guidelines (Lang RM, et al., 2015). Exclusively tests of excellent technical quality were used in the study. Echocardiographic examinations were carried out by the same expert operator to minimize measurement errors. However, the operator was not aware of the patient’s clinical data and the values represented the average of at least three measurements. The left ventricular ejection fraction (LVEF) was calculated by the Simpson biplane method according to the following formula: LVEF = [left ventricular end-diastolic volume (LVEDV)-LV end-systolic volume (LVESV)/LVEDV * 100 as mean of two measures in four and two apical chambers. Both volumes were subsequently indexed for body surface area (BSA) and expressed in ml/m^2^. Cardiac output has been calculated as a continuity equation, and the cardiac index (CI) expressed in ml/min/m^2^ by means of continuity equation and the dP/dT as parameters of global systolic left ventricular function, as suggested by the guidelines (Lang RM, et al., 2015). Left atrial volume (LAV) was measured with the area-length method and indexed for BSA (LAVI). Diastolic dysfunction was assessed by recording pulse-wave Doppler patterns at the mitral, in order to obtain early (E) and late (A) diastolic filling velocities from the 4-chamber view. Tissue Doppler imaging was performed to evaluate septal E′ and the E/E′ ratio was also calculated (Nagueh SF, et al., 2016).

Right ventricular systolic parameters were also estimated, assessed by calculating the tricuspid annulus plane systolic excursion (TAPSE) and the systolic pulmonary artery pressure (s-PAP) estimate. The TAPSE was assessed using the M-Mode on the tricuspidal ring and expresses the longitudinal systolic function of shortening the right ventricle, a parameter assessed on the basis of ventricular interdependence. The diameter and collapsibility of the inferior vena cava (IVC) during the inhalation-expiratory phase in subcostal projection was used to estimate of the right atrial pressure. Tricuspid regurgitant velocity (TRV) was obtained by continuous Doppler at the level of the atrioventricular plane of the tricuspid valve in projection with the four apical chambers or, in the case of eccentric jets, in parasternal short axis: therefore the s-PAP was derived through the Bernoulli equation: s-PAP = 4 (TRVpeak)^2^ + Right atrial pressure (RAP). The evaluation of the diameter of the outflow tract of the right ventricle (RVOT) was assessed in the long axis parasternal projection. The area of the right atrium (RAA) was evaluated in apical four chambers projection (Lang RM, et al., 2015). For speckle tracking analysis digital loops were captured, recording at least three consecutive beats, and analysed off-line using a dedicated software (EchoPAC 20.0; GE Medical Systems, Milwaukee, United States) by two operators who were blinded to the clinical characteristics of the patients. The same operators derived bidimensional, Doppler and speckle tracking parameters according to the most recent recommendations. If the software was not able to assess a segment due to poor image quality after manual correction of endocardial border, the segment considered as inadequate was excluded from the analysis. Briefly, each ventricular wall was analyzed into three segments with a total of 17 segments for the whole myocardium. Longitudinal strain was calculated for each segment, considering the higher value; thus the global longitudinal strain (GLS) was obtained as the mean of all 17 segments (Badano LP, et al., 2018).

### Laboratory Determinations

All laboratory measurements were performed after a minimum fasting period of 12 h on peripheral blood samples. Serum creatinine was assayed by the Roche Creatinine Plus assay (Homan-La Roche, Basel, Switzerland) on a clinical chemistry analyzer (Roche/Hitachi modular analysis system, module P), renal function was then calculated by e-GFR according to the equation suggested by the Chronic Kidney disease Epidemiology Collaborating Group (CKD-EPI). Serum sodium and potassium levels were measured by indirect potentiometry (Cobas, Roche). NT-proBNP values were assessed by enzyme-linked immunosorbent assay (Elecsys proBNP assay, Roche Diagnostics).

### Statistical Analysis

Continuous variables were summarized as mean and standard deviation (SD) (normally distributed data) or as median and interquartile range (IQR) (non-normally distributed data), these latter presented graphically by box and wisher plot. Categorical data were expressed as percentages. The evolution of therapies across time was tested by Cochran’s Q Test. The longitudinal changes of the key variables during the follow-up were analysed by the linear mixed model (LMM). All variables which deviate from the normal distribution were log-transformed (ln) before to be introduced into LMM. Multifactorial hypotheses were tested by multiple LMM, a statistic technique which allows to deal with 300 repeated observations over time in 60 patients (i.e., 5 repeated measurements per patient). In the multiple model of CI we adjusted for BMI, e-GFR, LVEDV/BSA, E/e’, and GLS. In this model, data were expressed as regression coefficient, 95% confident interval (95% CI) and *p*-value. Data analyses were performed by a commercially available statistical software (SPSS version 22 for Windows (Chicago, Illinois, United States) and STATA statistical package (version 13, Texas, United States).

## Results

Of the 60 outpatients evaluated, 87% were males and 25% active smokers. NYHA class II was represented in 65% and NYHA class III in 35% of patients.

At baseline, the e-GFR mean values were 69 ± 18 ml/min/1.73 m^2^ and BMI was 31 ± 5 kg/m^2^. BP values were 120 ± 12 mmHg for systolic and 74 ± 8 mmHg for diastolic component, respectively. The median value of NT-proBNP was 1,172 (800–1904) pg/ml and the mean values for EF and CI were 32.3 ± 1.6% and 1764.8 ± 211.7 ml/min/m^2^, respectively. The remaining baseline clinical, biochemical and echocardiographic patients’ characteristics are reported in table 1. The main aetiologies for HF were ischemic heart disease in (55%) and arterial hypertension (41%). Considering the associated comorbidities, 40% of patients showed chronic obstructive pulmonary disease COPD, 65% had type 2 diabetes mellitus (T2DM), 83% dyslipidaemia, 30% atrial fibrillation and 47% of patients renal dysfunction. Finally, 50% of patients had an electronic device [implantable cardioverter defibrillator (ICD) or cardiac resynchronization therapy defibrillator (CRTd)]. In particular, about this point, all patients, had previously been implanted at least 12 months before the beginning of sac/val treatment. In addition, patients who met the indication for CRTd or ICD implantation during follow-up study were not considered for data analysis. However, none of the enrolled patients met this indication.

**TABLE 1:** Baseline characteristics of patients that completed the study.

	Whole population (N = 60)
*Demographic and clinical parameters*	
Age, *years*	67 ± 11
Gender (males), *%*	87
BMI, *kg/m* ^*2*^	31 ± 5
Smokers, *%*	25
Ischemic Heart disease, *%*	55
COPD, *%*	40
T2DM, *%*	65
Dyslipidemia, *%*	83
Atrial Fibrillation, *%*	30
Renal Dysfunction, *%*	47
Arterial Hypertension, *%*	41
Systolic BP, *mmHg*	120 ± 12
Diastolic BP, *mmHg*	74 ± 8
Heart rate, *beats/min*	65 ± 8
Respiratory rate, *breath/min*	17 ± 3
MLHFQ, *total score*	90 ± 4
*Biochemical parameters*	
Na, *mmol/l*	140.9 ± 2.3
K, *mmol/l*	4.4 ± 0.4
Creatinine, *mg/dl*	1.1 ± 0.3
e-GFR, *ml/min/1.73m* ^*2*^	69 ± 18
NT- proBNP, *pg/ml*	1,172 (800–1904)
*Echocardiographic parameters*	
LAVI, *ml/m* ^*2*^	45.1 ± 12.0
LVEDV/BSA, *ml/m* ^*2*^	85.1 ± 11.1
LVESV/BSA, *ml/m* ^*2*^	57.6 ± 7.5
LVEF, *%*	32.3 ± 1.6
Cardiac index, *ml/min/m* ^*2*^	1764.8 ± 211.7
E/A	0.65 ± 0.14
E/e’	17 (15–18)
GLS, *%*	−8.0 (from −9.4 to −7.0)
RVOT, *cm*	2.9 ± 0.5
RA area, *cm* ^*2*^	20.1 ± 2.9
TAPSE, *mm*	16.6 ± 1.5
s-PAP, *mmHg*	45.1 ± 8.2
IVC, *mm*	19.5 (19.4–19.6)

BMI, body mass index; BP, blood pressure; COPD, chronic obstructive pulmonary disease; T2DM, type 2 diabetes mellitus; MLHFQ, Minnesota Living with Heart Failure Questionnaire; HOMA, homeostatic model assessment; e-GFR, estimated glomerular filtration rate; NT-proBNP, N-terminal pro-brain natriuretic peptide; LAVI, left atrial volume index; LVEDV/BSA, left ventricular end-diastolic volume index/body surface area; LVESV/BSA, left ventricular end-systolic volume index/body surface area; LVEF, left ventricular ejection fraction; GLS, global longitudinal strain; RVOT, Right Ventricular Outflow Tract; RA, Right Atrium; TAPSE, Tricuspid annular plane systolic excursion; s-PAP, systolic pulmonary arterial pressure; ICV, inferior vena cava**.**

At baseline, 74% of the population started the lowest dose of sac/val (24/26) and 26% of the patients the intermediate dose (49/51). After a 24 months of follow-up, there was a significant improvement of the functional status, thus 34% of patients shifted to NYHA class I (*p* < 0.0001) whereas 50% was in NYHA class II and 16% in NYHA class III. Of the initial sixty patients, one died for lung cancer and two patients (3.33%) were lost at follow-up. Only two patients showed an acute HF exacerbation during follow-up, which needed a re-hospitalization. At 24-months of follow-up, 41% of patients was taking the lowest dose of sac/val (24/26), 38% of the patients the intermediate dose (49/51) and 21% the highest dose (97/103). Regarding adverse events, there were only two episodes of symptomatic hypotension, which did not lead to discontinuation of treatment.

### Evolution of Pharmacological Treatment Over Time

Main drug treatments at baseline and during follow-up are illustrated in Table 2. Before the switch to sac/val, 80% of patients were treated with angiotensin-converting enzyme inhibitors (ACEs) and 20% with angiotensin receptor blockers (ARBs). At baseline, 48% of patients were taking mineral receptor antagonists (MRAs), 98% beta-blockers and 97% of patients were treated with loop diuretics. Moreover, 70% of the patients were taking statins, 29% oral anticoagulant (OAC) and 55% antiplatelet drugs. Across time, patients taking loop diuretics decreased significantly, according with this after 24 months only 82% of them took loop diuretics recording a reduction of 15% (*p* < 0.0001) also patients taking MRA significantly decreased reporting a reduction of 14% (*p* = 0.01). The use of statins, betablockers, OAC, antiplatelet agents remained unchanged throughout the follow-up period. About diabetic patients, none of them were treated with glucagon-like peptide 1 receptor agonists (GLP-1 RA) therapy and only four (10.2%) patients were treated with sodium glucose cotransporter two inhibitors (SGLT2i).

**TABLE 2:** Evolution of therapies across time.

	Time (months)					
—	0 (%)	6 (%)	12 (%)	18 (%)	24 (%)	p*
MRAs	48	41	39	37	36	0.01
Statins	70	70	70	70	70	1.00
Beta-blockers	98	98	98	98	98	1.00
OACs	29	29	29	29	29	1.00
Antiplatelet therapy	55	55	55	55	55	1.00
Loop Diuretics	97	89	86	84	82	<0.001

p* derived by Test di Cochran’ Q on listwise.

MRAs, mineralocorticoid receptor antagonists; OACs, oral anticoagulants.

### Evolution of Study Biomarkers Over Time

**FIGURE 1:**
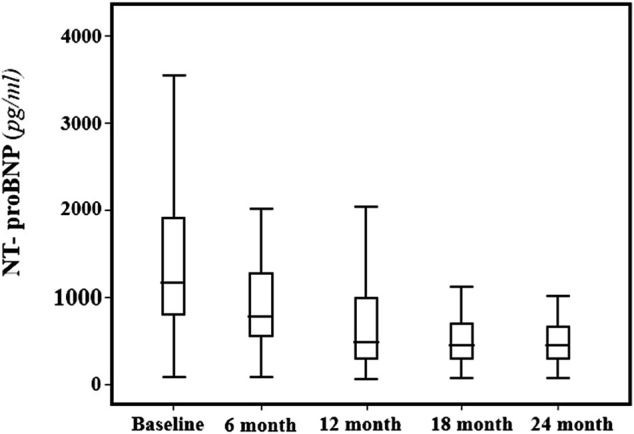
NTproBNP (pg/ml) median and interquartile range values at baseline and every 6 months, during the 24-months follow-up.

**FIGURE 2:**
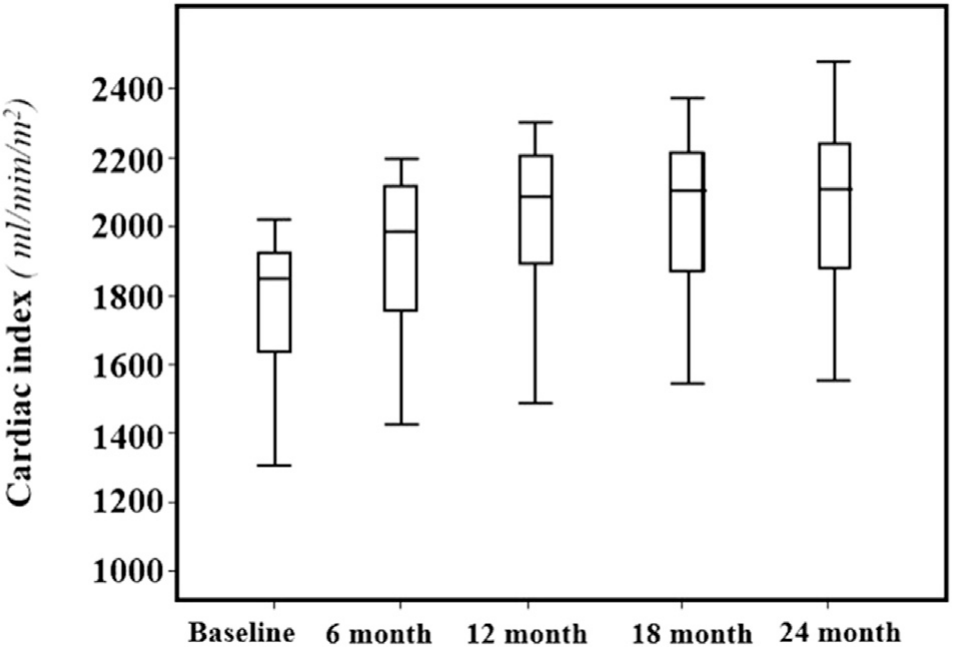
Mean values and standard deviation of the Cardiac Index (ml/min/m^2^) at baseline and every 6 months, during the 24-months follow-up.

Across the follow-up period both clinical, hemodynamic and biochemical parameters significantly improved. In particular, MLHFQ total score (from 90 ± 4 to 74 ± 4, *p* < 0.001), BMI (31 ± 5 vs. 29 ± 4 kg/m^2^, *p* = 0.001), respiratory rate (17 ± 3 vs. 13 ± 2 breath/min, *p* < 0.001), creatinine (from 1.12 ± 0.26 to 0.87 ± 0.18 mg/dl, *p* < 0.001), sodium (140.9 ± 2.3 vs. 137.7 ± 1.4 mmol/L, *p* < 0.0001) and NT-ProBNP (from 1,172 (800–1904) to 450 (296–721) pg/ml, *p* < 0.001, Figure 1), were significantly reduced. On the contrary, e-GFR (from 69 ± 18 to 85 ± 16 ml/min/m2) and potassium (4.42 ± 0.38 vs. 5.05 ± 0.25 mmol/L, *p* < 0.0001) significantly increased. Similarly, also the echocardiographic parameters significantly changed. According with this LAVI (45.1 ± 12.0 vs. 37.6 ± 9.9 ml/m^2^, *p* < 0.001), LVEDVI (85.1 ± 11.1 vs. 78.9 ± 7.3 ml/min/m^2^, *p* = 0.019), LVESVI (57.61 ± 7.50 vs. 52.4 ± 7.1 ml/min/m^2^, *p* = 0.005), and right heart parameters so as RVOT (2.9 ± 0.5 vs. 2.05 ± 0.4 cm, *p* = 0.004) and RA area (20.14 ± 2.91 vs. 16.95 ± 2.13 cm^2^, *p* < 0.001) significantly reduced over time. Moreover, all the echocardiographic indicators of left and right ventricular systolic function, in particular, GLS (median: 8.0, IQR: from −9.4 to −7.0 vs. median: 13.4, IQR: from −14.5 to −12.3%, *p* < 0.001), LVEF (32.30 ± 1.60 vs. 37.00 ± 1.69%, *p* < 0.001), cardiac index (from 1764.8 ± 211.8 to 2042.8 ± 257.9 ml/min/m^2^, *p* < 0.001, Figure 2) and TAPSE (16.57 ± 1.47 vs. 19.5 ± 2.61, *p* < 0.001), were significant improved. In addition, also diastolic parameters so as E/A (0.65 ± 0.14 vs. 0.75 ± 0.14, *p* < 0.001) and E/e’ [median: 17, IQR: from 15 to 18 vs. median 14.00, IQR: from 12 to 15.8, *p* < 0.001] showed a significant improvement.

Finally, also ICV diameter was significantly reduced (median: 19.5 mm, IQR: from 19.4 to 19.6 vs. median: 18 mm, IQR from 17 to 19, *p* = 0.001) with a consensual reduction in s-PAP (45.07 ± 8.17 vs. 33.1 ± 6.01 mmHg, *p* = 0.001). Systolic and diastolic BP and heart rate did not modify significantly.

The average changes and the 95% CIs of repeated measurements of study biomarkers associated to a fixed increase (6 months) in time are given in table 3. In detail, the mean reduction per semester of variables which significantly changed over time were as follows: 4.236 for MLHFQ (*p* < 0.001), −0.587 kg/m^2^ for BMI (*p* = 0.001), −1.268 breath/min for Respiratory rate(*p* < 0.001), −0.066 mg/dl for Creatinine (*p* = 0.001), -0.77 pg/ml NT-proBNP (*p* < 0.001), −0.918 mmol/L for Na, LAVI (*p* < 0.001), −1.058 ml/m^2^ for LVEDVI (*p* = 0.019), −1.056 ml/m^2^ for LVESVI (*p* = 0.005), −0.95 for E/e’ (*p* < 0.001), −1.274% for GLS, −0.185 cm^2^ for RVOT (*p* = 0.004), −0.799 cm^2^ for RA area (*p* < 0.001), −2.71 mm Hg for s-PAP (*p* < 0.001), and −0.98 mm for ICV (*p* = 0.001). An opposite pattern, i.e., a significant increase overtime was found for the following variables: a 4.719 ml/min/1.73 m^2^ e-GFR (*p* < 0.001), 0.165 mmol/L for K (*p* < 0.001), 1.11% for LVEF (*p* < 0.001), 67.024 ml/min/m^2^ for CI (*p* < 0.001), 0.03 for E/A (*p* < 0.001), and 0.656 mm for TAPSE (*p* < 0.001). No significant changes over time were observed in BP and heart rate (*p* > 0.527) (Table 3).

**TABLE 3:** Univariate linear mixed models of study variables over time.

	Regression coefficient (95%CI)	*p*-value
*Clinical parameters*		
MLHFQ, *total score*	−4.236 (from −5.783 to −2.689)	<0.001
BMI, *kg/m* ^*2*^	−0.587 (from −0.944 to −0.23)	0.001
Systolic BP, *mmHg*	−0.194 (from −1.325 to 0.936)	0.736
Diastolic BP, *mmHg*	0.077 (from −0.566–0.719)	0.815
Heart rate, *beats/min*	−0.28 (from −1.148 to 0.588)	0.527
Respiratory rate, *breath/min*	−1.268 (from −1.813 to −0.722)	<0.001
*Biochemical parameters*		
Creatinine, *mg/dl*	−0.066 (from −0.089 to −0.044)	<0.001
e-GFR, *ml/min/1.73m* ^*2*^	4.719 (from 3.247 to 6.191)	<0.001
NT- proBNP^a^, *pg/ml*	−0.77 (from −0.84 to −0.71)	<0.001
Na, *mmol/l*	−0.918 (from −1,243 to −0.594)	<0.001
K, *mmol/l*	0.165 (from 0.106 to 0.224)	<0.001
*Echocardiographic parameters*		
LAVI, *ml/m* ^*2*^	−1.803 (from −2.674 to −0.933)	<0.001
LVEDVI, *ml/m* ^*2*^	−1.058 (from −1.942 to −0.175)	0.019
LVESVI, *ml/m* ^*2*^	−1.056 (from −1.786 to −0.326)	0.005
LVEF, *%*	1.11 (from 0.616 to 1.604)	<0.001
Cardiac index, *ml/min/m* ^*2*^	67.024 (from 40.491 to 93.558)	<0.001
E/A	0.03 (from 0.015 to 0.045)	<0.001
E/e’^a^	−0.95 (from −0.97 to −0.92)	<0.001
GLS^a^, *%*	−1.274 (from −1.641 to −0.907)	<0.001
RVOT, *cm*	−0.185 (from −0.31 to −0.06)	0.004
RA area, *cm* ^*2*^	−0.799 (from −1.127 to −0.47)	<0.001
TAPSE, *mm*	0.656 (from 0.336 to 0.976)	<0.001
s-PAP, *mmHg*	−2.71 (from −4.244 to −1.177)	0.001
ICV^a^, *mm*	−0.98 (from −0.99 to −0.97)	0.001

Data are regression coefficient, 95% CI and *p* values.

a Log-transformed (ln) variables.

BMI, body mass index; BP, blood pressure; MLHFQ, Minnesota Living with Heart Failure Questionnaire; HOMA, homeostatic model assessment; e-GFR, estimated glomerular filtration rate; NT-proBNP, N-terminal pro-brain natriuretic peptide; LAVI, left atrial volume index; LVEDVI, left ventricular end-diastolic volume index; LVESVI, left ventricular end-systolic volume index; LVEF, left ventricular ejection fraction; GLS, global longitudinal strain; RVOT, Right Ventricular Outflow Tract; RA, Right Atrium; TAPSE, Tricuspid annular plane systolic excursion; s-PAP, systolic pulmonary arterial pressure; ICV, inferior cave vein.

### Independent Correlates of Repeated Measurements of Cardiac Index

To assess the independent correlates of CI over time, a multiple linear mixed model including a series of covariates was fitted, in particular HF risk factors and HF medications balanced for the number of patients in the study population. In this model, the longitudinal evolutions of CI were associated to concomitant changes of GLS and E/e’ ratio and were independent of the other covariates (Table 4). To put this in perspective, 1 unit increase in E/e’ and 1% increase in GLS values, indicating a clinically significantly worsening of diastolic and systolic function, were independently associated to −12.79 (*p* < 0.001) and −2.15 (*p* = 0.005), reduction in CI over time, respectively.

**TABLE 4:** Multivariate linear mixed models of Cardiac Index over time.

	Regression coefficient (95%CI)	*p*-value
BMI, kg/m^2^	−3.71 (from −11.89 to 4.48)	0.372
e-GFR, *ml/min/1.73m* ^*2*^	0.82 (from −0.4 to 2.03)	0.187
LVEDV/BSA, *ml/m* ^*2*^	−0.11 (from −1.04 to 0.81)	0.804
E/e’	−12.79 (from −19.14 to −6.45)	<0.001
GLS, *%*	−2.15 (from −3.6 to −0.7)	0.005
Loop diuretics	17.99 (from −21.01 to 57)	0.362
MRAs	−21.94 (from −63.13 to 19.25)	0.293
Beta blockers	87.62 (from −409.01 to 584.24)	0.724
Age	−0.48 (from −6.69 to 5.72)	0.876
Gender	−83.24 (from −269.89 to 103.41)	0.374
Diabetes	88.33 (from −37.76 to 214.42)	0.165
COPD	13.26 (from −118.28 to 144.79)	0.840
Arterial Hypertension	20.44 (from −124.36 to 165.24)	0.778
Ischemic heart disease	−17.07 (from −165.19 to 131.05)	0.818

BMI, body mass index; e-GFR, estimated glomerular filtration rate; LVEDV/BSA, left ventricular end-diastolic volume index/body surface area; GLS, global longitudinal strain; MRAs, mineral receptor antagonists COPD, chronic obstructive pulmonary disease.

## Discussion

In this longitudinal-observational, one-center study, including 300 repeated observations over time in 60 HFrEF symptomatic patients despite optimal medical therapy, sac/val treatment showed effectiveness, safety and effect durability every 6 months up to 2 years of follow-up with significant improvement of several clinical, hemodynamic and echocardiographic parameters. Patients’ clinical conditions had substantially improved with reduction of MLHFQ score and improvement of NYHA class. Together with this, also BMI and RR significantly reduced without significant changes in BP and heart rate, indicating the reduction of systemic congestion with haemodynamic conditions improvement, without an excessively hypotensive effect. According with this, only two episodes of symptomatic hypotension occurred without the need for treatment discontinuation.

The QoL improvement with sac/val treatment is an important issue and, according with this, in PARADIGM-HF it occurred early after 8 months as demonstrated by increase in KCCQ (McMurray JJ, et al., 2014) and this finding has been also reported in different real life settings so as Parasail study and Provide-HF (Haddad H, et al., 2020; Mentz RJ, et al., 2020) after a follow-up of 12 weeks and 12 months, respectively. Our study shows more consistent data because the QoL improvement rises up at 6 months by the start of the treatment with sac/val and it persists over 24 months.

Another additional favourable effect of sac/val treatment was represented by improvement of LV remodelling and betterment of right cardiac structure and function parameters. According with this, the sac/val treatment was associated with reduction in LAVI and end-systolic and end-diastolic LV volumes, together with this also diastolic function parameters were improved, with E/A ratio increase and E/e’ reduction, thus showing a lowering of intraventricular filling pressure. LV contractility also became better, as demonstrated by the significant change in GLS values and LVEF. Moreover, the longitudinal improvements of CI were associated to positive changes of GLS and E/e’ ratio.

Of interest, also right heart parameters significantly improved with reduction of right chambers enlargement (RVOT and RA area), increase in right ventricle systolic performance as demonstrated by higher values of TAPSE. Obviously, the reverse myocardial remodelling may justify the reduction in NT-proBNP levels that indicates a better hemodynamic condition as demonstrating by the lowering of s-PAP and IVC diameter, together with a significant reduction of diuretic therapy, indicating a significant improvement of both pulmonary and systemic congestion.

Finally, another important evidence from the study is that also renal function was significantly improved as demonstrated by reduction of creatinine levels and increase in e-GFR values, this effect already evident at an early stage, persisted for 2 years. As pharmacological effect, sodium serum levels significantly reduced and serum potassium increased, however remaining always in the normal range, thus confirming the safety of treatment.

The positive effect of sac/val on clinical, biochemical and echocardiographic parameters may be justified by the particular pharmacological strategy of sac/val, with the simultaneous blocking of both RAAS and neprilysin systems. Inhibition of neprilysin results in increased natriuretic peptides levels with positive effects on sodium and water balance, arterial blood pressure and sympathetic modulation, in particular natriuresis and vasodilatation (Gu J, et al., 2010; Singh et al., 2017). On the other hand, the inhibition of angiotensin-2 effects allows to exercise antiproliferative effects protecting from hypertrophy and fibrosis at different sites so as myocardium and kidney. As previously reported, the great clinical benefit of sac/val has been demonstrated in the PARADIGM-HF trial (McMurray JJ, et al., 2014), however the positive effect of sac/val treatment in clinical practice is remarked, so as in our study, by the reduction in NT-proBNP levels, an important marker of cardiac remodelling, volume overload and haemodynamic instability. Moreover, as reported by Jannuzzi et al. in the PROVE-HF study, the reduction in NT-proBNP levels in HFrEF patients following treatment with sac/val was associated, after 12 months, with a positive change of several cardiac remodelling measures (Januzzi JL Jr, et al., 2019).

It’s known that cardiac remodelling, consisting in geometric and functional LV changes leading to systolic performance reduction, represents the main mechanism of HF progression and it is associated with a worse clinical prognosis including death and hospitalization for HF (Udelson JE, Konstam MA, 2011; Kemp CD, Conte JV, 2012). The potential reverse remodelling effect of sac/val has been demonstrated in the PRIME study, a prospective randomized trial showing that sac/val is more effective than the only angiotensin receptor blocker to improve functional mitral regurgitation HF associated after 12 months of follow-up, without significant changes in LVEF (Kang DH, et al., 2019). Moreover, several real world evidence studies have reported an improvement of cardiac remodelling markers and patients’ functional capacity (Spannella F, et al., 2019; Moliner-Abós C, et al., 2019; Wachter R, et al., 2019; Haddad H, et al., 2020; Mentz RJ, et al., 2020; Correale M, et al., 2020; Cosentino et al., 2019; Martens P, et al., 2018). In particular, a retrospective cohort study on a small number of HFrEF patients showed, after a mean follow-up of 14 months, a significant improvement in LVEF and GLS (De Vecchis R, et al., 2019). Among prospective studies, short term evaluations demonstrated a significant improvement in LV remodelling markers, including a betterment of mitral filling pattern (Martens P, et al., 2018) together with clinical and functional parameters (Cosentino et al., 2019). Recently, Castrichini et al. demonstrated that the introduction of sac/val in HFrEF, despite a long history of disease, provides a global LV and LA reverse remodeling in more than 25% of cases, both at standard and advanced echocardiographic evaluations, after a median follow-up of 9 months. However, there was not significant improvement in right heart functional parameters (Castrichini M, et al., 2020). On the contrary, an observation from the Danua Heart Failure Registry reported that in HFrEF patients the treatment with sac/val improved not only LV echo parameters but also allowed an increase in TAPSE and decrease in s-PAP, thus indicating a positive effect on right ventricle function, however the analysis was limited to 12 months without any information on a longer observation (Correale M, et al., 2020). In comparison with previous real world evidence (RWE) studies, our investigation demonstrates that the average changes of repeated measurements of both clinical and echocardiographic parameters associated to a fixed increase in time (every 6-months) were statistically significant. In particular, the parameters’ improvement was already evident at 6 months but it was more pronounced at the long term. This is the first study with a follow-up to 24 months, demonstrating the long-term safety and tolerability of sac/val treatment with durability of its positive effects on clinical conditions, LV remodelling and right heart structure and function.

About the nephroprotective effect of sac/val, it had been demonstrated both in clinical trials (McMurray JJ, et al., 2014; Damman K, et al., 2018) and in real life setting (Spannella F, et al., 2019). According with this, Spannella et al. reported a significant improvement of renal function after 12 months of follow-up in HFrEF patients treated with sac/val despite a reduction in SBP and slightly increase in LVEF, without significant changes in diuretic therapy (Spannella F, et al., 2019).

In our study, the improvement of renal function is already significant after 6 months, moreover the average changes of e-GFR are maintained statistically significant until 2 years. It’s plausible that the improvement in cardiac remodelling and haemodynamic conditions may favour a better renal blood flow maintaining effective renal haemodynamic and consequently promoting glomerular filtration rate, an effect amplified by blocking the RAAS. This is demonstrated in our study by the increase in BP and reduction in diuretic therapy. On the other hand, the reduction of diuretic therapy after sac/val treatment was reported both in the PARADIGM-HF study and in real life setting (Vardeny O, et al., 2019; Wachter R, et al., 2019).

However, natriuretic peptides may positively affect different target organs and in particular the kidney, where natriuretic peptides receptors are mainly expressed. According with this, in experimental studies natriuretic peptides exercise positive effects on sodium proximal tubular reabsorption and tubuloglomerular feedback, these actions together with angiotensin-2 blockage may amplify the anti-inflammatory and antifibrotic effects (Judge P, et al., 2015; Jing W, et al., 2017). These mechanisms could explain the long-term nephroprotective effect as observed in our study and, because renal function has an important prognostic role in HF patients, all these findings are clinically relevant (Damman K, et al., 2014).

Some limitations need to be addressed. At first, this is not a randomized trial and we have not a matched control group. However, as each patient before the enrollment was treated with the best possible therapy, according to current guidelines, but still symptomatic.

Moreover, considering that no other intervention was allowed in the study, in particular the ICD/CRTd implantation, the long-term changes of study variables, can be attributed to sac/val. According with this, a recent work has shown that, in patients with HFrEF the CRTd, and particularly in failing heart patients with T2DM, could affect the arrhythmic burden, hospitalizations for HF, and CRTd responders rate. (Sardu C, et al., 2018). However, as reported, none of the patients underwent to ICD/CRTd implantation during the follow-up avoiding a possible confounding effect on study results.

Other limitations are represented by the relatively small population and the lack of cardiac magnetic resonance (CMR) parameters for the enrolled patients, and CMR represents a well validated method to better characterize the myocardial tissue in particular to detect LV fibrosis. However, a point of strength is that this is a RWE study considering clinically more complex patients, with comorbidities and polypharmacy in comparison with RCTs.

## Conclusion

Our study strengthens scientific evidence of treatment with sac/val in HFrEF patients. It provides a complete and detailed demonstration of the treatment’s positive effects, demonstrating the significant improvement of hemodynamic, clinical, biochemical and both standard and advanced echocardiographic parameters of left and right chambers. All these findings are observed during a 2-year follow-up and through a 6-monthly control of the study variables. To the best of our knowledge, this real-life investigation is the only 24 months follow up study demonstrating the efficacy and durability of sac/val treatment in HFrEF patients for the whole duration of follow-up and without major adverse events.

Considering all positive effects also in a real life setting and the safety and durability of the intervention, present data support the need to start treatment with sac/val as soon as possible, and to provide future trials to investigate the cost effectiveness of sac/val in optimizing HF treatment in particular before ICD implantation. An early approach in HFrEF patients would allow to improve, not only the QoL, but also to act on the pathophysiological mechanisms of the disease, promoting reverse cardiac remodelling, preserving renal function and thus allowing a positive and substantial intervention on the prognosis. It is plausible that larger prospective registers and observational studies considering propensity score matched populations will be able to answer the many open questions.

## Data Availability

Deidentified data will be made available upon request to readers for verification purposes.
