# Supplementary material for: Impact of Sacubitril/Valsartan on Clinical and Echocardiographic Parameters in Heart Failure Patients With Reduced Ejection Fraction: Data From a Real Life 2-year Follow-Up Study
Source: Front Pharmacol. 2021 Aug 16;12:733475. doi: 10.3389/fphar.2021.733475 (PMC8415264; doi:10.3389/fphar.2021.733475)
Supplement: Supplementary file 1 [file Table1.DOCX]

**Table 1 – Supplementary material - Baseline and follow-up characteristics of patients who completed the study**

|  | **Whole population baseline** | **Follow-up** | **p value** |
| --- | --- | --- | --- |
| ***Demographic and clinical parameters*** | |  |  |
| BMI, *Kg/m^2^* | 31±5 | 29±4 | 0.001 |
| Systolic BP, *mmHg* | 120±12 | 117±8 | 0.736 |
| Diastolic BP, *mmHg* | 74±8 | 73±6 | 0.815 |
| Heart rate, *beats/min* | 65±8 | 63±6 | 0.527 |
| Respiratory rate, *breath/min* | 17±3 | 13±2 | <0.001 |
| MLHFQ, *total score* | 90±4 | 74±4 | <0.001 |
| ***Biochemical parameters*** |  |  |  |
| Na, *mmol/l* | 140.9±2.3 | 137.7±1.4 | <0.0001 |
| K, *mmol/l* | 4.4±0.4 | 5.05±0.25 | <0.0001 |
| Creatinine, *mg/dl* | 1.1±0.3 | 0.87±0.18 | <0.001 |
| e-GFR, *ml/min/1.73m^2^* | 69±18 | 85±16 | <0.0001 |
| NT- proBNP, *pg/ml* | 1172 (800-1904) | 450 (296-721) | <0.001 |
| ***Echocardiographic parameters*** | |  |  |
| LAVI, *ml/m^2^* | 45.1±12.0 | 37.6±9.9 | <0.001 |
| LVEDV/BSA, *ml/m^2^* | 85.1±11.1 | 78.9±7.3 | 0.019 |
| LVESV/BSA, *ml/m^2^* | 57.6±7.5 | 52.4±7.1 | 0.005 |
| LVEF, *%* | 32.3±1.6 | 37.00±1.69 | <0.001 |
| Cardiac index, *ml/min/m^2^* | 1764.8±211.7 | 2042.8±257.9 | <0.001 |
| E/A | 0.65±0.14 | 0.75±0.14 | <0.001 |
| E/e’ | 17 (15-18) | 14.00 (12-15.8) | <0.001 |
| GLS, *%* | -8.0 (-9.4 -7.0) | -13.4 (-14.5 / -12.3) | <0.001 |
| RVOT, *cm* | 2.9±0.5 | 2.05±0.4 | 0.004 |
| RA area, *cm^2^* | 20.1±2.9 | 16.95±2.13 | <0.001 |
| TAPSE, *mm* | 16.6±1.5 | 19.5±2.61 | <0.001 |
| s-PAP, *mmHg* | 45.1±8.2 | 33.1±6.01 | 0.001 |
| IVC, *mm* | 19.5 (19.4-19.6) | 18 (17-19) | 0.001 |
